# Musculoskeletal Pain as a Marker of Health Quality. Findings from the Epidemiological Sleep Study among the Adult Population of São Paulo City

**DOI:** 10.1371/journal.pone.0142726

**Published:** 2015-11-24

**Authors:** Suely Roizenblatt, Altay L. Souza, Luciana Palombini, Luciana M. Godoy, Sergio Tufik, Lia Rita A. Bittencourt

**Affiliations:** 1 Department of Internal Medicine, UNIFESP, São Paulo, Brazil; 2 Department of Psychobiology, UNIFESP, São Paulo, Brazil; University of Rome Tor Vergata, ITALY

## Abstract

**Background:**

We are witnessing the growth of urban populations, particularly in the developing world. São Paulo, the largest city in South America, continues to grow, and this growth is dramatically effecting the environment and human health. The aim of this study was to estimate the point prevalence of chronic pain in São Paulo city dwellers and to explore the influence of aspects related to urbanicity.

**Methods:**

A two-stage cluster randomized sample included 1100 individuals of the city of Sao Paulo, representing the population proportionally in terms of gender, age and social classes in 2007. For this observational cross-sectional study, the household sample was interviewed using validated questionnaires for sociodemographic aspects, the Beck inventories for anxiety and depression, the WHOQoL-REF for quality of life, the Chalder Fatigue Scale. Musculoskeletal pain was defined as diffuse pain or pain located in the back, joints or limbs. Data regarding sleep complaints and polysomnography were obtained from the Epidemiologic Sleep Study conducted in São Paulo city in 2007.

**Results:**

The prevalence estimate of chronic musculoskeletal pain was approximately 27%, with a female/male ratio of approximately 2.6/1. The predictors were being in the age-range of 30–39 years, low socioeconomic and schooling levels, obesity, sedentarism, fatigue, non-restorative sleep, daytime sleepiness, poor sleep quality, poor life quality, anxiety and depression symptoms. Psychological wellbeing was the main discriminator between responders with chronic musculoskeletal pain and the controls, followed by depression for the participants with poor psychological wellbeing, and fatigue, for the remaining ones. Insomnia syndrome was the third-level discriminator for those with fatigue, whereas sleep quality for those without fatigue.

**Conclusions:**

Musculoskeletal pain was frequently reported by São Paulo city dwellers and its correlates with psychological and sleep aspects are suggestive of a response to urbanicity.

**Trial Registration:**

ClinicalTrials.gov NCT00596713

## Introduction

With the recent increases in urbanization, particularly between 1997 and 2007, the high reported prevalences of mental and sleep disorders have attracted much interest to Sao Paulo city as a model of the urbanicity burst that has occurred in developing countries.[1–5]

Increases in sleep complaints[2] and the consequent increases in the demand for medical services[3] have been evidenced by the last Epidemiologic Sleep Study which was conducted in Sao Paulo city (EPISONO) in 2007. This population-based study estimated prevalences greater than 30% for both obstructive sleep apnea (OSA)[4] and insomnia[5] and demonstrated associations of sleep disorders with cardiovascular risks[6], obesity[7], and mood disorders.[5]

Similar to disordered sleep, chronic pain, allied to accumulation of stressful events, physical and mental co-morbidity,[8, 9] affect life quality and increase the risk of all-cause mortality.[8] Chronic musculoskeletal pain is the most frequent modality of chronic pain, and its association with impaired sleep has been recognized.[10]

The lack of interest in subjects who are not affected to a significant degree has been implicated in the underestimation of pain as a multidimensional condition[11–14] with biological, psychological, and social causes and consequences.[15]

Sleep impairments are considered more reliable predictors of pain than vice versa,[16] and thus far, little attention has been devoted to pain complaints according to sleep related conditions in urban situations. With the hypothesis that chronic musculoskeletal pain may result from the impact of living in a large and overcrowded city, we attempted to fill the gap in knowledge concerning its prevalence in São Paulo city, and the extent to which it is influenced by subjective and objective indicators of impaired sleep. Additionally, we examined the associations of chronic musculoskeletal pain with socio-demographic factors, life style, life quality and mood.

## Sampling and Methods

In this population-based cross-sectional survey, we analyzed secondary data derived from the EPISONO dataset, which was designed to evaluate the prevalence of sleep disorders and their risk factors. A three-stage-cluster sampling technique with unequal selection probabilities was used to obtain a representative sample in terms of gender, adult age, and socioeconomic status.[17] For this single-center study a sample size of 1,101 individuals was established to allow prevalence estimates with 3% precision. Consenting subjects to undergo polysomnography (PSG) were 1.042 and the refusal rate was 5.4% (59 volunteers). Details about EPISONO methodology can be found in our previous publications.[4, 17]

The questions used to screen for the presence of chronic musculoskeletal pain were worded as follows: “Have you felt body aches during the last 6 months?” When the answer was “generally yes” a question about pain topography was applied. The respondents who characterized their pain as located in the back, joints, or limbs, or as diffuse were included in this survey and formed the group of individuals with chronic musculoskeletal pain (MP group)

The study was approved by the local Ethical Committee (CEP-0593/06) and registered with ClinicalTrials.gov (Identifier-NCT00596713), URL: http://www.clinicaltrials.gov/ct2/show/NCT00596713?term=NCT00596713&rank=1).

Portuguese-validated questionnaires were administered in the participant’s homes and at the Sleep Laboratory. We used data from the following home inventories: Brazilian Socioeconomic and Demographic inventory,[18] ‘‘UNIFESP” Sleep Questionnaire,[19] and Pittsburgh Sleep Quality Index (PSQI). [20] In addition, we established a cut-off of 9 years for years of schooling. In the Sleep Laboratory, we assessed health and sleep information using the local standardized inventory,[21] and administered the International Physical Activity Questionnaire version-6, [22]the Beck Inventories for Anxiety and Depression,[23] the World Health Organization Quality of Life brief version (WHOQoL-BREF),[24] 11-item-Chalder Fatigue Scale,[25] and the Epworth Sleepiness Scale. [26]

Full-night PSG (EMBLA S7000^TM^; Embla Systems, CO, USA) was conducted in the Sleep Laboratory. Methodologies for sleep recording, and the criteria used to diagnose sleep disorders are described in our previous publications.[4, 5]

For the diagnosis of Insomnia syndrome, the presence of PSG criteria in combination with at least one of the sleep related complaints proposed by DSM-IV was required. Based on PSG, insomnia was defined by the presence of at least one of the following findings: Sleep latency > 30 minutes, WASO > 30 minutes, TST < 360 minutes, and terminal wakefulness > 30 minutes. DSM-IV insomnia was diagnosed in the presence of self-reported difficulty in initiating sleep ≥ 30 minutes, difficulty in maintaining sleep (waking ≥ 3 or being awake ≥ 30 minutes), or early morning awakenings, occurring at least 3 times per week, lasting > 1 month. The association of symptoms with non-restorative sleep, despitesufficient opportunity for sleep was also required. [5]

### Statistics

The data regarding anthropometric and sociodemographic profile, sedentarism (physical activity), fatigue, quality of life, mood, sleep-related symptoms, and sleep recording parameters were described using the weighted mean [standard error (SE)] or weighted percentage and standard error [%(SE)]. Prevalence was estimated using pseudo-likelihood maximization when stratifying and weighing the sample to adequately represent the population. Effect sizes of the measurements [odds ratios (OR) and 95% Confidence intervals (95%CI)] was chosen to compare groups.

The exploratory analyses used to identify factors associated with pain were adjusted for sex, age, and body mass index (BMI, except when these parameters were analyzed) and ANCOVA, to explore the differences between groups in terms of age, BMI, quality of life, anxiety and depression scores, and subjective and objective sleep data.

Chi-square tests were applied to assess the associations of pain with the anthropometric and sociodemographic data, frequency of sedentarism, fatigue, and sleep-related symptoms, and insomnia. For these analyses, questionnaire scores were categorized according to the following cut-off values: Chalder Fatigue Scale ≥4, Epworth Sleepiness Scale >9, and PSQI ≥5.

**Fig 1:**
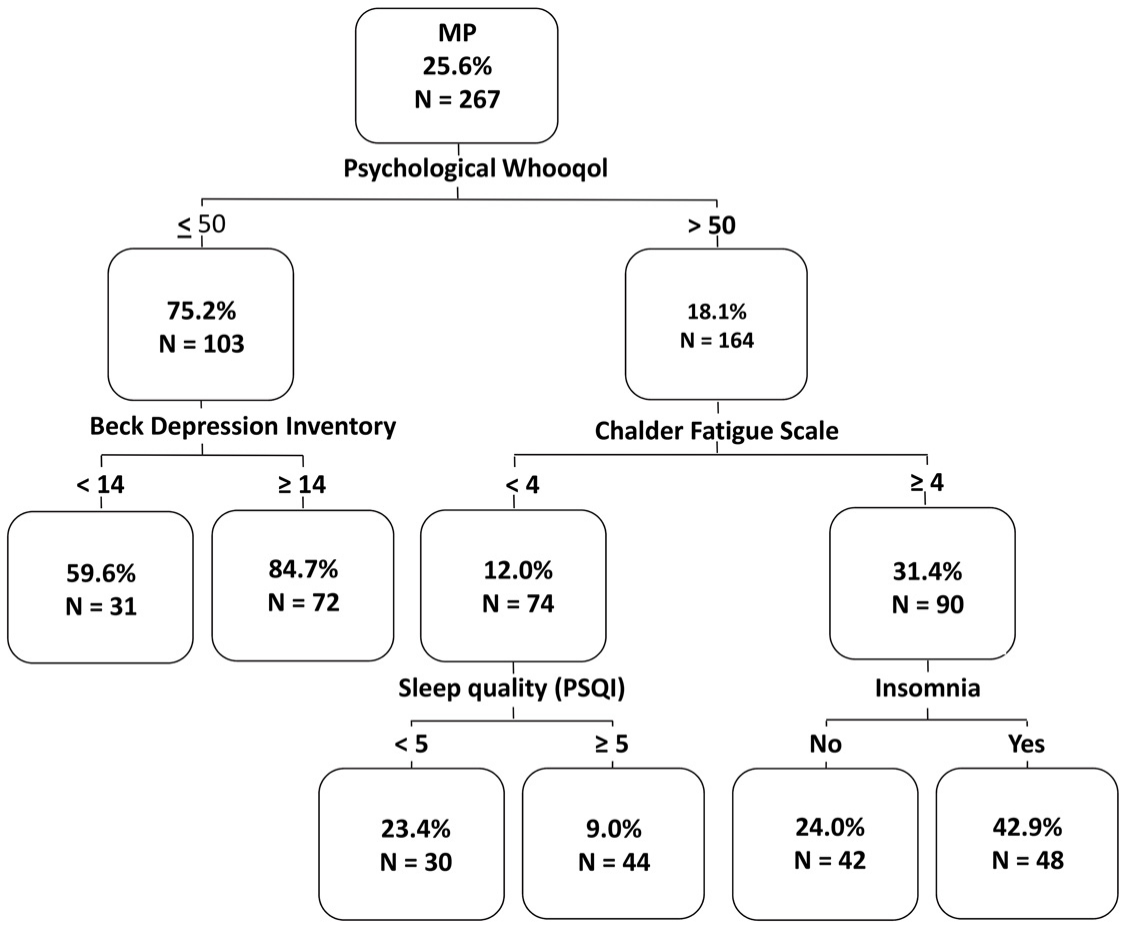
Discriminators between responders with musculoskeletal pain (MP group) and controls. Percentage of pain individuals in each node of CHAID Tree Analysis.

We fitted binary logistic regression to investigate the parameters that might influence the pain condition. The outcome was pain and the explanatory variables were anthropometric data, sociodemographic parameters, life style factors, the questionnaires data, and the objective sleep parameters. Adjusted OR and 95%CI for controls vs. MP group are presented. Finally, a tree analysis via the CHAID method was used to define a hierarchical model of all relevant variables (Fig 1).

The analyses were performed using SPSS version 18.0 (IBM Corp^TM^) and the adopted level of significance was p<0.05.

## Results

The prevalence estimates of chronic musculoskeletal pain were 26.83% (SE = 1.30) overall, 34.85% (SE = 2.56) among the females and 15.88% (SE = 1.99), among the males (p<0.01), and there was no difference in mean age between control and MP groups [mean (SE) = 41.31 (1.02) vs. 43.05 (1.22) years]. However, the age range of 30–39 years was associated with pain, as shown in Table 1, which presents the characteristics of the groups.

**Table 1:** Sociodemographic characteristics of the population. Weighed frequencies and standard errors (SE). Odds ratios adjusted for age, sex, and body mass index (BMI).

		Controls N = 774		Chronic musculoskeletal pain N = 268		Controls vs. Chronic musculoskeletal pain	
		%	SE	%	SE	OR	95%CI
Gender	Women	46.84%	3.25%	71.40%	3.82%	3.41	2.11–5.51
	Men	53.16%	3.25%	28.60%	3.82%	1.00	-
Age (years)	20–29	28.06%	2.23%	18.63%	3.24%	1.00	-
	30–39	21.52%	1.87%	29.68%	3.99%	3.19	1.89–5.39
	40–49	21.99%	1.46%	20.83%	3.54%	1.50	0.76–2.94
	50–59	14.60%	1.78%	18.71%	3.02%	1.75	0.85–3.59
	60–80	13.83%	2.11%	12.15%	2.90%	1.29	0.63–2.60
BMI (kg/m^2^)	<25	25.45%	2.69%	16.67%	3.12%	1.00	-
	25 to 30	48.71%	2.75%	40.46%	4.52%	1.07	0.58–1.97
	> = 30	25.84%	2.24%	42.87%	4.53%	2.25	1.27–3.98
Economic status	A	12.64%	1.77%	5.99%	1.86%	1.00	-
	B	42.39%	2.51%	35.46%	3.58%	1.70	0.90–3.25
	C	37.29%	2.78%	50.58%	3.82%	2.38	1.22–4.62
	D and E	7.68%	1.43%	7.97%	1.88%	1.72	0.70–4.26
Schooling	<9 years	32.66%	2.30%	51.12%	4.54%	1.70	1.03–2.79
Sedentarism		62.30%	2.51%	74.29%	2.89%	1.98	1.32–2.96

Compared to controls, the MP group scored higher on the Chalder fatigue Scale [mean (SE) = 3.16 (0.14) vs.5.88 (0.23), respectively, p<0.001], the Epworth Sleepiness Scale [mean (SE) = 7.99 (0.24) vs. 8.96 (0.35), respectively, p = 0.04], and the PSQI, denoting poorer sleep quality [mean (SE) = 8.58 (0.25) vs. 5.18 (0.17), respectively, p<0.001]. Table 2 displays the associations of pain with sleep related factors.

**Table 2:** Complaints associated with disordered sleep Weighed frequencies and standard errors (SE). Odds ratios adjusted for age, sex, and body mass index (BMI). Fatigue (Chalder score ≥4), daytime sleepiness (Epworth Score >9), poor sleep quality (PSQI score≥5).

	Controls N = 774		Chronic musculoskeletal pain N = 268		Controls vs.Chronic musculoskeletal pain	
	%	SE	%	SE	OR	95%CI
Morning headache	31.69%	3.29%	64.29%	3.79%	3.58	2.10–6.08
Fatigue	29.11%	2.00%	66.76%	3.67%	4.70	2.92–7.58
Non-restorative sleep	37.11%	2.47%	81.67%	2.27%	7.13	4.41–11.52
Daytime sleepiness	34.11%	2.25%	45.27%	3.92%	1.42	1.01–1.99
Poor sleep quality	23.02%	1.85%	60.01%	3.24%	3.66	2.33–5.74
Insomnia syndrome	9.04%	1.40%	31.47%	3.55%	3.80	2.34–6.22

Regarding mood, the MP group scored higher than the controls on the BAI [mean (SE) = 14.39 (0.91) vs. 6.14 (0.31), respectively, p<0.001], and the BDI [mean (SE) = 14.08 (0.75) vs. 8.25 (0.41), respectively, p<0.001], which suggests association of mild anxiety and depression symptoms with pain (Table 3). In terms of wellbeing, the MP group scored lower than did the controls in the total WHOQoL-BREF [mean (SE) = 56.01 (1.00) vs. 68.08 (0.62), respectively p<0.001] and its domains. The mean and SE scores of the MP group vs. controls, were as follows: in the physical domain 57.86 (1.24) and 69.46 (0.57), respectively, p<0.001; in the psychological domain, 52.25 (1.10) and 74.40 (0.67), respectively, p<0.001; in the social domain 64.22 (1.82) and 70.55 (0,84), respectively, p<0.001, and in the environmental domain, 49.70 (0.95) and 57.92 (0.81), respectively, p<0.001.

**Table 3:** Mood and life quality inventories Weighed frequencies and standard errors (SE). Odds ratios adjusted for age, sex, and body mass index (BMI).

	Controls N = 774		Chronic musculoskeletal pain N = 268		controls vs.Chronic musculoskeletal pain	
	mean	SE	mean	SE	OR	95%CI
Beck depression inventory	8.07	0.43	13.80	0.84	1.10	1.06–1.14
Beck anxiety inventory	5.86	0.38	14.43	1.11	1.13	1.1–1.16
Total WHOQoL-BREF	68.14	0.58	56.90	0.92	0.90	0.89–0.92
Physical WHOQoL-BREF	74.28	0.66	52.42	1.34	0.89	0.88–0.91
Psychological WHOQoL-BREF	69.29	0.60	59.44	1.29	0.95	0.93–0.96
Social WHOQoL-BREF	70.63	0.85	65.50	1.46	0.98	0.97–0.99
Environmental WHOQoL-BREF	58.39	0.82	50.25	1.07	0.96	0.94–0.97

There was no significant difference between the MP group and the controls with respect to the weighted prevalence of individuals who subjectively reported sleeping less than 7 hours per night 4 days per week [55.60% (SE = 3.80%) in MP group and 42,20% (SE = 2.40%) in controls, p = 0.07]. Similarly, the weighted prevalences of individuals with recorded total sleep time below 7 hours were not different between groups [mean (SE) = 336,52 (7.68) in MP group and 343,96 (3.00) in controls, p = 0.90], and no significant difference in the other PSG parameters were detected.

Tree analysis was performed to detect the best discriminators between MP and controls (Fig 1). The psychological domain of the WHOQoL-BREF was found to be the first-level discriminator (chi-square = 203.28, p<0.001) followed by the BDI for those with low scorings (chi-square = 10.88, p = 0.02), and the Chalder Fatigue Scale for those with high scores (chi-square = 49.63, p<0.001). A third-level of discrimination was observed in terms of sleep quality (PSQI) for those with fatigue scoring ≥ 4 (chi-square = 20.13, p<0.001), and insomnia syndrome for those with low fatigue scores (chi-square = 11.28, p<0.01).

## Discussion

The prevalence estimate of musculoskeletal pain in this representative cross-sectional household sample of Sao Paulo city dwellers, was approximately 27%. The predictors of musculoskeletal pain were being in the age-range of 30 and 39 years, obesity, belonging to socioeconomic class C, sedentarism, fatigue, non-restorative sleep, daytime sleepiness, poor sleep quality, anxiety and depression symptoms, and poor life quality in the four domains of WHOQoL-BREF.

In contrast with the reported prevalence of about 20% commonly accepted for chronic pain,[9, 12, 27] the greater prevalence observed here may be attributable to the restriction of the present study to a large city population. Regardless, our estimate is similar to those that have been reported in developing countries.[28] The poor scores of the MP group in the environmental domain (WHOQoL-BREF) are suggestive of the influence of urban sprawl on pain, in terms of lack of safety/security, health/social care, information, recreation, accessibility, and also exposition to pollution, noise, traffic, and financial problems. Indeed, our findings support the associations of pain conditions with poor socioeconomic status and low education levels, as previously reported[28, 29] in the context of the increases in inequality that accompany the urbanization process.[30]

Nevertheless, the main discriminators between participants with chronic musculoskeletal pain and the remaining ones were of psychological and sleep nature, particularly the psychological wellbeing, depression, fatigue, insomnia syndrome and sleep quality. Life quality in patients with chronic pain has been considered an important measure in health care[9] and is associated with sleep quality and mental health.[31] The high prevalences of sleep complaints,[2] sleep disorders,[4, 5] and the links of impaired sleep with physical[6, 7] and mental health[5] reported in the EPISONO, and the current study indicate the common points that are shared by disordered sleep and musculoskeletal pain in the urban conditions of Sao Paulo city. Moreover, morning headache and fatigue, which have been attributed to impaired sleep and negative mood status,[31] were also associated with musculoskeletal pain.

Exposed to the stressful process of urbanization, people tend to live their everyday lives without being health conscious,[1] which causes negative mental health outcomes.[15] In this circumstance, mood factors have been associated with sedentarism, as previously reported in EPISONO, and our findings demonstrate that both contribute to musculoskeletal pain.

As described in the context of urbanization, sleep-deprived individuals are more prone to anxiety and depression symptoms[10, 31] Accordingly, we observed high frequency of sleep restriction ≤7 hours/night in all groups.[31] Considering that sleep restriction hampers endogenous pain inhibitory function,[32] the association of musculoskeletal pain with the productive age range corroborates with the concept of chronic musculoskeletal pain as a habituation of the body and mind to the distress of urban life.

The first limitation of our study is the absence of information regarding disability; however wellbeing cannot be sufficiently explained by functional abilities.[33] Second, we cannot rule out inaccuracies in the self-reported pain condition in isolated cases, but this issue was addressed by the population-based method of the study. Finally, neither the topography nor the intensity of pain were analyzed, due to the coexistence of pain in different body regions and site-to-site differences in the pain intensity of the same individual.

In contrast to other epidemiological studies on pain, the enrollment process for this research did not focus on pain, but instead, on EPISONO criteria.[17] Therefore, the possibility of a biased sampling of participants who were motivated by their pain condition was mitigated. Population-based studies of individuals with disordered sleep who are affected by musculoskeletal pain are scarce[34, 35] and this research sheds light on the roles of subjective and objective indicators of impaired sleep as risk factors for chronic musculoskeletal pain in individuals who are exposed to urbanicity. Our data reinforce the individual-level modifiable aspects that are involved in urban life, such as socio-cultural demands, sedentarism, and also sleep health.

In conclusion, musculoskeletal pain affects approximately one of every four dwellers of Sao Paulo city and may represent a boundary between health and disease. This is an issue of public health concern and is related to the process of urbanization and overcrowding in one of the major cities of the globe.

## Abbreviations

EPISONO: Epidemiologic Sleep Study Among the Adult Population of São Paulo City
MP group: the group of individuals with chronic musculoskeletal pain
OSA: obstructive sleep apnea
PSG: polysomnography ()
PSQI: Pittsburgh Sleep Quality Index (
WHOQoL-BREF: World Health Organization Quality of Life brief version
