# Correction: Musculoskeletal Pain as a Marker of Health Quality. Findings from the Epidemiological Sleep Study among the Adult Population of São Paulo City

**DOI:** 10.1371/journal.pone.0150330

**Published:** 2016-02-22

**Authors:** Suely Roizenblatt, Altay L. Souza, Luciana Palombini, Luciana M. Godoy, Sergio Tufik, Lia Rita A. Bittencourt

The DOI provided in the Data Availability statement is in incorrect. The correct DOI is: http://dx.doi.org/10.5061/dryad.h3k43.
